# Assessment of Myosteatosis on Computed Tomography by Automatic Generation of a Muscle Quality Map Using a Web-Based Toolkit: Feasibility Study

**DOI:** 10.2196/23049

**Published:** 2020-10-19

**Authors:** Dong Wook Kim, Kyung Won Kim, Yousun Ko, Taeyong Park, Seungwoo Khang, Heeryeol Jeong, Kyoyeong Koo, Jeongjin Lee, Hong-Kyu Kim, Jiyeon Ha, Yu Sub Sung, Youngbin Shin

**Affiliations:** 1 Department of Radiology and Research Institute of Radiology Asan Medical Center Seoul Republic of Korea; 2 Biomedical Research Center Asan Medical Center Seoul Republic of Korea; 3 School of Computer Science and Engineering Soongsil University Seoul Republic of Korea; 4 Health Screening and Promotion Center Asan Medical Center Seoul Republic of Korea; 5 Clinical Research Center Asan Medical Center Seoul Republic of Korea; 6 Department of Convergence Medicine University of Ulsan College of Medicine Seoul Republic of Korea

**Keywords:** body composition, muscle, skeletal, sarcopenia, computed tomography, x-ray, scan, web-based tool, feasibility, automated, CT

## Abstract

**Background:**

Muscle quality is associated with fatty degeneration or infiltration of the muscle, which may be associated with decreased muscle function and increased disability.

**Objective:**

The aim of this study is to evaluate the feasibility of automated quantitative measurements of the skeletal muscle on computed tomography (CT) images to assess normal-attenuation muscle and myosteatosis.

**Methods:**

We developed a web-based toolkit to generate a muscle quality map by categorizing muscle components. First, automatic segmentation of the total abdominal muscle area (TAMA), visceral fat area, and subcutaneous fat area was performed using a predeveloped deep learning model on a single axial CT image at the L3 vertebral level. Second, the Hounsfield unit of each pixel in the TAMA was measured and categorized into 3 components: normal-attenuation muscle area (NAMA), low-attenuation muscle area (LAMA), and inter/intramuscular adipose tissue (IMAT) area. The myosteatosis area was derived by adding the LAMA and IMAT area. We tested the feasibility of the toolkit using randomly selected healthy participants, comprising 6 different age groups (20 to 79 years). With stratification by sex, these indices were compared between age groups using 1-way analysis of variance (ANOVA). Correlations between the myosteatosis area or muscle densities and fat areas were analyzed using Pearson correlation coefficient r.

**Results:**

A total of 240 healthy participants (135 men and 105 women) with 40 participants per age group were included in the study. In the 1-way ANOVA, the NAMA, LAMA, and IMAT were significantly different between the age groups in both male and female participants (*P*≤.004), whereas the TAMA showed a significant difference only in male participants (male, *P*<.001; female, *P*=.88). The myosteatosis area had a strong negative correlation with muscle densities (*r*=–0.833 to –0.894), a moderate positive correlation with visceral fat areas (*r*=0.607 to 0.669), and a weak positive correlation with the subcutaneous fat areas (*r*=0.305 to 0.441).

**Conclusions:**

The automated web-based toolkit is feasible and enables quantitative CT assessment of myosteatosis, which can be a potential quantitative biomarker for evaluating structural and functional changes brought on by aging in the skeletal muscle.

## Introduction

Measurement of muscle is one of the fastest growing research areas in medicine, as quantity and quality of muscle in individuals are reportedly associated with morbidity and mortality in various diseases [1,2]. Muscle mass is frequently assessed using medical imaging such as computed tomography (CT) or magnetic resonance imaging (MRI) [3]. However, an issue of discrepancy between the muscle mass measured on imaging and muscle functions, such as strength and mobility, has been raised. Therefore, the assessment of muscle quality by imaging is gaining a focus in the research and clinical diagnoses of relevant diseases. Specifically, the CT density or attenuation can be quantified using a standardized unit (ie, Hounsfield unit [HU]), enabling a standardized muscle quality evaluation on CT.

Recent studies demonstrated that muscle quality is associated with fatty degeneration or fatty infiltration of the muscle (ie, myosteatosis), which may be associated with decreased muscle function and increased disability [4]. Based on the imaging characteristics in CT, fat can be stored as follows: (1) in the intermuscular adipose tissue, which is observed as gross fat between muscle groups; (2) in the intramuscular adipose tissue as extramyocellular lipids, which are denoted as gross fat tissues between muscle fibers in the same muscle group; and (3) in the intramyocellular lipid droplets, which are not visually demonstrated as fat but are indicated by decreased muscle density on CT [5]. It is known that the intermuscular and intramuscular adipose tissues (IMAT) can be depicted as areas with gross fat density of –190 HU to –30 HU, whereas the intramyocellular lipid can be reflected as low-attenuation muscle area (LAMA) with –29 HU to +29 HU within the muscle [6,7]. The healthy muscle without myosteatosis is observed as normal-attenuation muscle area (NAMA) with +30 HU to +150 HU [6].

The muscle density at the L3 vertebral level has been used for muscle quality evaluation on abdominal CT in majority of prior studies because it easily measures muscle density [1,8]. Recent advancements in technology evaluate myosteatosis based on the distribution and amount of IMAT and LAMA. In this paper, we evaluate the feasibility of fully automated quantitative measurement of the skeletal muscle on CT using a web-based toolkit for assessing normal-attenuation muscle and myosteatosis.

## Methods

This study, which uses retrospective data, was approved by the institutional review board of Asan Medical Center, and a waiver for written informed consent was obtained.

### Generation of a Muscle Quality Map

Among the abdominal CT images, a single axial image at the inferior endplate level of the L3 vertebra at the portal venous phase was selected for image assessment [9]. The selected CT image was saved as a digital imaging and communications in medicine (DICOM) file and was uploaded to our web-based toolkit in a drag-and-drop manner. First, we performed automatic segmentation of the abdominal compartments using a predeveloped deep learning model based on a fully convolutional network [10]. This model was reported to segment abdominal body compartments into the total abdominal muscle area (TAMA), subcutaneous fat area, and visceral fat area with a dice similarity coefficient of 0.97 and mean cross-sectional area error of 2.26% [10].

**Figure 1:**
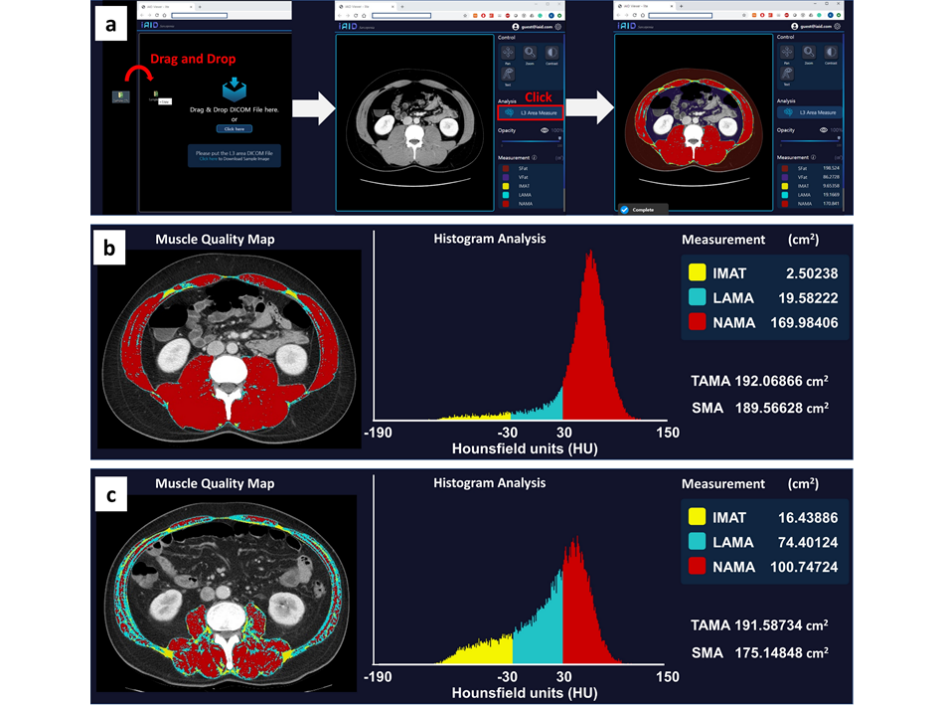
Muscle quality map generation using an automated web-based toolkit. IMAT: inter/intramuscular adipose tissue area; LAMA: low-attenuation muscle area; NAMA: normal-attenuation muscle area; SMA: skeletal muscle area; TAMA: total abdominal muscle area.

Second, we performed postprocessing to divide the TAMA into 3 muscle components using the pixel-wise measurement of CT density with the following range of HU for each component [6,7]: NAMA with +30 HU to +150 HU, LAMA with –29 HU to +29 HU, and IMAT with –190 HU to –30 HU. Subsequently, a muscle quality map was generated by combining NAMA, LAMA, and IMAT, which were displayed in different colors on the web-based toolkit (Figure 1A), enabling the quantitative assessment of muscle properties based on histogram analysis. For example, the participants with similar TAMA values can be differentiated into participants with healthy muscles (ie, mostly composed of NAMA) and those with fatty degenerated muscles (ie, comprising several LAMA and IMAT). The technique has been packaged and can be accessed at an iAID Viewer repository [11]. Figure 1 shows the process and examples of muscle quality map generation using the web-based iAID toolkit. A CT image is uploaded to the toolkit in a drag-and-drop manner. Muscle quality map and its measurement values are displayed upon clicking on the “L3 Area Measure” button (Figure 1A). Muscle quality maps and a histogram for a 28-year-old man with higher quality muscle are shown in Figure 1B; and muscle quality maps and a histogram for a 72-year-old man with lower quality muscle are shown in Figure 1C.

The quality of the generated muscle quality map was evaluated by a board-certified abdominal radiologist by comparing the original CT image and the muscle quality map image. If the segmentation quality was not acceptable, manual adjustment was performed using a stand-alone software.

### Feasibility Study in Healthy Participants

From the electronic database of Asan Medical Center, we retrospectively identified 3928 abdominal CT scans from healthy participants who had no disease or history of previous treatments and had undergone CT for medical checkup or workup for liver donation during the period from January 2008 to June 2016. We used the block randomization technique to randomly select 240 healthy participants (135 men and 105 women) belonging to 6 different age groups, from 20 to 79 years, with 10-year intervals (40 participants per age group).

CT was performed using a 16-channel (LightSpeed 16, GE Healthcare; Somatom Sensation 16, Siemens Medical Solution), a 64-channel (Discovery CT 750 HD, GE Healthcare; Somatom Definition AS, Siemens Medical Solution), or a 128-channel (Somatom Definition Flash, Siemens Medical Solution) CT scanner with the following parameters: 120 kVp, 200–220 mAs (maximum tube current with automated dose modulation), 1.5-5 mm section thickness and intervals, and a pitch of 0.6-1. Contrast agents were administered at a rate of 3-4 mL/s, and CT images (regardless of the CT protocols) were obtained, including the portal venous phase (65-72 seconds after contrast agent injection) in the craniocaudal direction.

### Statistical Analysis

To eliminate effects by different sexes, participants within each subgroup were stratified by sex. The mean values of muscle components (TAMA, NAMA, LAMA, and IMAT) were compared among the age groups using 1-way analysis of variance. A *P*<.05 was considered statistically significant. Correlation between the mean density of TAMA or skeletal muscle area (SMA; ie, NAMA + LAMA) and muscle components related to the adipose tissue (ie, LAMA, IMAT, or myosteatosis area represented by LAMA + IMAT) were calculated using Pearson correlation (absolute magnitude of Pearson correlation coefficient *r*=0-0.1, negligible correlation; *r*=0.1-0.4, weak correlation; *r*=0.4-0.7, moderate correlation; *r*=0.7-0.9, strong correlation; and *r*=0.9-1, very strong correlation) [12]. In addition, to investigate the correlation between the adipose tissue within and outside the muscle compartment, the correlation between the aforementioned muscle components related to the adipose tissue—LAMA, IMAT, and myosteatosis area—and the visceral and subcutaneous fat area was also analyzed using Pearson correlation. All statistical analyses were performed using the SPSS, version 21 (IBM Corp).

## Results

The characteristics of the 240 participants are presented in Table 1. In male participants, height and weight were significantly different across age groups (*P*<.001). In female participants, height was significantly different across age groups (*P*<.001). Body mass index was not significantly different both in male (*P*=.11) and female participants (*P*=.13).

**Table 1:** Characteristics of the study population.

Characteristics		Age group, years							*P* value
		20-29	30-39	40-49	50-59	60-69	70-79		
Participants, n		40	40	40	40	40	40	N/A^a^	
Age (years), mean (SD)		25.1 (2.83)	34.5 (2.39)	45.2 (2.93)	54.2 (2.63)	62.5 (2.62)	72.8 (2.35)	N/A^a^	
Male:female ratio		29:11	17:23	22:18	15:25	25:15	27:13	.007	
**Height (m), mean (SD)**		1.71 (0.08)	1.65 (0.10)	1.67 (0.09)	1.59 (0.07)	1.62 (0.08)	1.63 (0.09)	<.001	
	Male	1.74 (0.06)	1.74 (0.06)	1.73 (0.06)	1.67 (0.04)	1.67 (0.04)	1.68 (0.05)	<.001	
	Female	1.64 (0.07)	1.59 (0.06)	1.59 (0.05)	1.55 (0.04)	1.54 (0.04)	1.53 (0.05)	<.001	
**Weight (kg), mean (SD)**		69.8 (14.9)	66.8 (14.5)	67.5 (11.7)	62.3 (7.8)	63.3 (9.5)	62.8 (10.0)	.02	
	Male	74.2 (14.7)	78.9 (12.7)	73.4 (10.9)	69.8 (4.0)	67.2 (9.0)	65.7 (8.9)	.001	
	Female	58.3 (7.2)	57.8 (7.7)	60.3 (8.1)	57.8 (5.8)	56.8 (6.4)	56.8 (9.7)	.78	
**BMI (kg/m^2^), mean (SD)**		23.6 (4.0)	24.3 (3.4)	24.2 (3.2)	24.5 (2.2)	24.0 (2.6)	23.5 (3.0)	.65	
	Male	24.3 (4.3)	26.1 (3.6)	24.7 (3.6)	25.1 (2.0)	24.1 (2.7)	23.2 (2.5)	.11	
	Female	21.7 (2.2)	23.0 (2.5)	23.7 (2.6)	24.2 (2.2)	24.0 (2.6)	24.2 (3.9)	.13	
**Cause of CT^b^ examination, n (%)**									<.001
	Medical checkup	27 (67.5)	15 (37.5)	15 (37.5)	20 (50.0)	31 (77.5)	39 (97.5)		
	Workup for liver donation	13 (32.5)	25 (67.5)	25 (62.5)	20 (50.0)	9 (22.5)	1 (2.5)		

^a^N/A: not applicable.

^b^CT: computed tomography.

**Figure 2:**
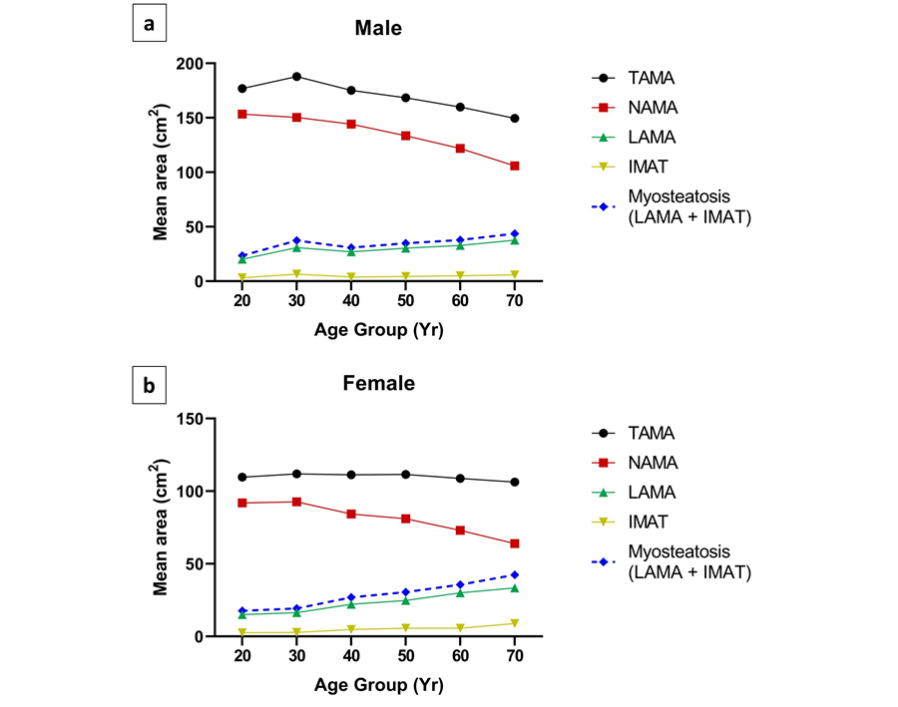
Area of muscle components according to the age groups in male and female participants. IMAT: inter/intramuscular adipose tissue area; LAMA: low-attenuation muscle area; NAMA: normal-attenuation muscle area; TAMA: total abdominal muscle area.

Figure 2 illustrates the mean areas of muscle components among different age groups of male and female participants.

The detailed information is presented in Table 2. TAMA showed a significant difference only in male participants (male, *P*<.001; female, *P*=.88). The age group of 30s had the highest TAMA (mean 187.9 cm^2^, SD 29.6 cm^2^) with a gradual decrease in area with aging in male participants. The TAMA in female participants did not differ significantly across the age groups. In contrast, NAMA, LAMA, and IMAT were significantly different among the age groups both in male and female participants (*P*<.001). In male participants, NAMA showed a gradual decrease with age (peak age: 20 years; mean 153.4 cm^2^, SD 20.0 cm^2^). In female participants, there was a peak in the 30s age group (mean 92.6 cm^2^, SD 15.1 cm^2^), followed by a gradual decrease in area with aging. The myosteatosis area showed a gradual increase with aging in female participants.

**Table 2:** Mean area and indices of muscle components in male and female participants.

Muscle components in participants		Age group, years						*P* value
		20-29	30-39	40-49	50-59	60-69	70-79	
**Male participants, n**		29	17	22	15	25	27	
	TAMA^a^, mean (SD)	177.0 (22.9)	187.9 (29.6)	175.3 (24.5)	168.4 (18.0)	160.0 (22.5)	149.6 (21.0)	<.001
	NAMA^b^, mean (SD)	153.4 (20.0)	150.4 (26.1)	144.3 (19.5)	133.5 (19.1)	122.0 (16.7)	105.9 (17.5)	<.001
	LAMA^c^, mean (SD)	20.2 (8.9)	30.9 (11.7)	27.0 (10.5)	30.5 (6.7)	32.9 (10.3)	37.8 (14.4)	<.001
	IMAT^d^, mean (SD)	3.3 (2.5)	6.6 (5.1)	3.9 (2.4)	4.4 (1.7)	5.1 (2.4)	5.9 (3.6)	.004
	Myosteatosis area (LAMA+IMAT), mean (SD)	23.5 (11.0)	37.5 (16.3)	31.0 (12.3)	34.9 (7.9)	38.0 (11.6)	43.7 (17.6)	<.001
**Female participants, n**		11	23	18	25	15	13	
	TAMA, mean (SD)	109.6 (10.9)	112.0 (15.3)	111.3 (11.1)	111.5 (15.4)	108.8 (13.8)	106.3 (17.2)	.88
	NAMA, mean (SD)	91.9 (9.5)	92.6 (15.1)	84.3 (11.2)	81.0 (13.1)	73.1 (14.2)	63.9 (17.3)	<.001
	LAMA, mean (SD)	15.1 (5.7)	16.5 (5.7)	22.1 (8.4)	24.9 (8.1)	30.0 (8.8)	33.5 (11.2)	<.001
	IMAT, mean (SD)	2.6 (1.4)	2.8 (1.6)	4.8 (3.1)	5.7 (2.5)	5.7 (3.3)	8.9 (5.1)	<.001
	Myosteatosis area (LAMA+IMAT), mean (SD)	17.7 (6.9)	19.3 (7.1)	27.0 (11.1)	30.5 (10.0)	35.7 (11.4)	42.4 (15.6)	<.001

^a^TAMA: total abdominal muscle area.

^b^NAMA: normal-attenuation muscle area.

^c^LAMA: low-attenuation muscle area.

^d^IMAT: inter/intramuscular adipose tissue area.

All muscle components related to the adipose tissue (ie, LAMA, IMAT, and myosteatosis area) had a moderate-to-strong negative correlation with the mean density of TAMA **(***r*=–0.629 to –0.884) and SMA (*r*=–0.647 to –0.898) (Table 3). They had a moderate positive correlation (*r*=0.474 to 0.686) with visceral fat area and weak-to-moderate positive correlation (*r*= 0.274 to 0.459) with subcutaneous fat area in both male and female participants (Table 3). In particular, LAMA showed higher correlation with visceral and subcutaneous fat compartments than that shown by IMAT. All the correlations in Table 3 are statistically significant (*P*<.05).

**Table 3:** Correlation between muscle components containing fat and muscle density and fat compartments.

Muscle components	Mean density of TAMA^a^		Mean density of SMA^b^ (NAMA^c^+LAMA^d^)			Visceral fat area			Subcutaneous fat area	
	Male	Female	Male	Female	Male		Female	Male		Female
LAMA	–0.845	–0.884	–0.847	–0.898	0.686		0.617	0.274		0.459
IMAT^e^	–0.629	–0.728	–0.647	–0.763	0.474		0.495	0.365		0.329
Myosteatosis area (LAMA+IMAT)	–0.833	–0.874	–0.838	–0.894	0.669		0.607	0.305		0.441

^a^TAMA: total abdominal muscle area.

^b^SMA: skeletal muscle area.

^c^NAMA: normal-attenuation muscle area.

^d^LAMA: low-attenuation muscle area.

^e^IMAT: inter/intramuscular adipose tissue area.

## Discussion

### Principal Results

We developed a web-based toolkit to generate a pixel-based automatic categorization of muscle components (ie, muscle quality map) within the generated segmented muscle compartment using predeveloped deep learning models. These muscle quality maps can illustrate spatial distribution of fat infiltration and provide insights into the muscle quality in individuals. In our study, NAMA gradually decreased with age; and LAMA and IMAT gradually increased with age. These results indicate that fat infiltration or fatty degeneration increases with aging. Therefore, NAMA might be an effective imaging biomarker for evaluation of muscle in individuals. Additionally, LAMA and IMAT might be used as biomarkers of myosteatosis and related diseases, such as metabolic syndrome [1,5].

### Comparison With Previous Work

Sarcopenia, which is defined as the loss of muscle mass or function, is associated with increased morbidity and mortality in various diseases [1,2]. Recently, the revised consensus of the European Working Group on Sarcopenia in Older People emphasized muscle strength and quality as a key characteristic of sarcopenia in the definition and diagnostic criteria of sarcopenia [8]. Accordingly, both qualitative and quantitative evaluations of muscle are highly recommended in sarcopenia diagnosis. Among the diagnostic tests for muscle quantity measurements, muscle quality evaluation can be performed only using cross-sectional imaging such as CT and MRI. The dual-energy x-ray absorptiometry and bioelectrical impedance analysis cannot differentiate healthy muscles from fatty degenerated muscles. Currently, the CT density or attenuation is well-calibrated and standardized (ie, HU of zero for water) across the CT acquisition protocols and imaging machines, whereas the signal intensity of MRI might differ between protocols and machines. Therefore, the muscle quality map on CT might be the optimal option to evaluate myosteatosis.

There are several software programs (both open source and licensed) that analyze muscle and fat composition in CT [13]. However, a software providing muscle quality maps at the L3 vertebral level is not yet available. We developed our own software for performing a fully automated segmentation of body compartments and generating a muscle quality map at the L3 vertebral level [11]. This software is publicly available for academic purposes. Considering the increasing recognition of myosteatosis and sarcopenia as prognostic biomarkers for various diseases in older patients, measuring muscle quality and muscle mass easily is highly recommended for researchers and clinicians.

### Limitations

Our study has some limitations. First, we tested the muscle quality map in a relatively small number of participants (N=240). We randomly assigned 40 participants into each age group; thus, our study is an experimental study rather than an epidemiologic study and may not reflect the real-world trends of muscle quality with aging. There was an imbalance between male and female participants, which may preclude the real-world data. In addition, we could not provide any criteria or cutoff value to diagnose myosteatosis. Therefore, an epidemiological study using large populations might be required. Second, the segmented areas of each muscle component might be affected by the different CT protocols and scanners. However, this is beyond the scope of the current study and needs to be evaluated in a separate study. Third, the general applicability of our toolkit should be validated in an external dataset, particularly because the predeveloped segmentation model was trained at our institution and could thus lead to overfitting.

### Conclusion

In conclusion, the automated web-based toolkit is feasible and enables a quantitative CT assessment of normal-attenuation muscle and myosteatosis, which can be effective quantitative biomarkers for evaluating structural and functional changes brought on by aging in the skeletal muscle.

## Abbreviations

ANOVA: analysis of variance
CT: computed tomography
DICOM: digital imaging and communications in medicine
HU: Hounsfield Unit
IMAT: inter/intramuscular adipose tissue
LAMA: low-attenuation muscle area
MRI: magnetic resonance imaging
NAMA: normal-attenuation muscle area
SMA: skeletal muscle area
TAMA: total abdominal muscle area
